# Loss Compensation and Coherent Virtual Absorption via Virtual Complex Frequency Wave Excitation

**DOI:** 10.1002/nap2.70125

**Published:** 2026-05-08

**Authors:** Sheng Zhang, Zhi Hong Hang

**Affiliations:** ^1^ School of Physical Science and Technology & Institute for Advanced Study Soochow University Suzhou China

**Keywords:** coherent virtual absorption, complex frequency wave, loss compensation

## Abstract

Complex frequency waves (CFWs), exhibiting extraordinary propagation properties including virtual gain/loss, have attracted increasing research attention. However, the experimental excitation of CFWs at high frequencies, especially in microwave and optical regimes, remains a big challenge. Here, by establishing a mathematical bridge between real and complex frequencies, this work presents a general method for CFW excitation, termed virtual CFW excitation, enabling us to obtain all electromagnetic responses for complex frequencies, including wavevector, electric/magnetic field, and transmission/reflection coefficient, even in the presence of intrinsic noise. Specifically, using virtual CFW excitation can offset the effect caused by material losses and the lost field information of both a point source and surface plasmon polaritons can be successfully recovered. Moreover, coherent virtual absorption is verified by proof‐of‐principle microwave transmission‐line experiments. Our finding provides an effective tool to explore phenomena of complex frequencies, mitigating experimental difficulties and promoting the future development of optical devices.

## Graphical abstract

By establishing a mathematical bridge between real and complex frequencies, we present a general method for complex frequency wave excitation to obtain all electromagnetic responses at complex frequencies. Using properties of complex frequency waves, we compensate for the propagating loss of point sources and surface plasmon polaritons. Moreover, coherent virtual absorption is experimentally verified in a microwave transmission line.

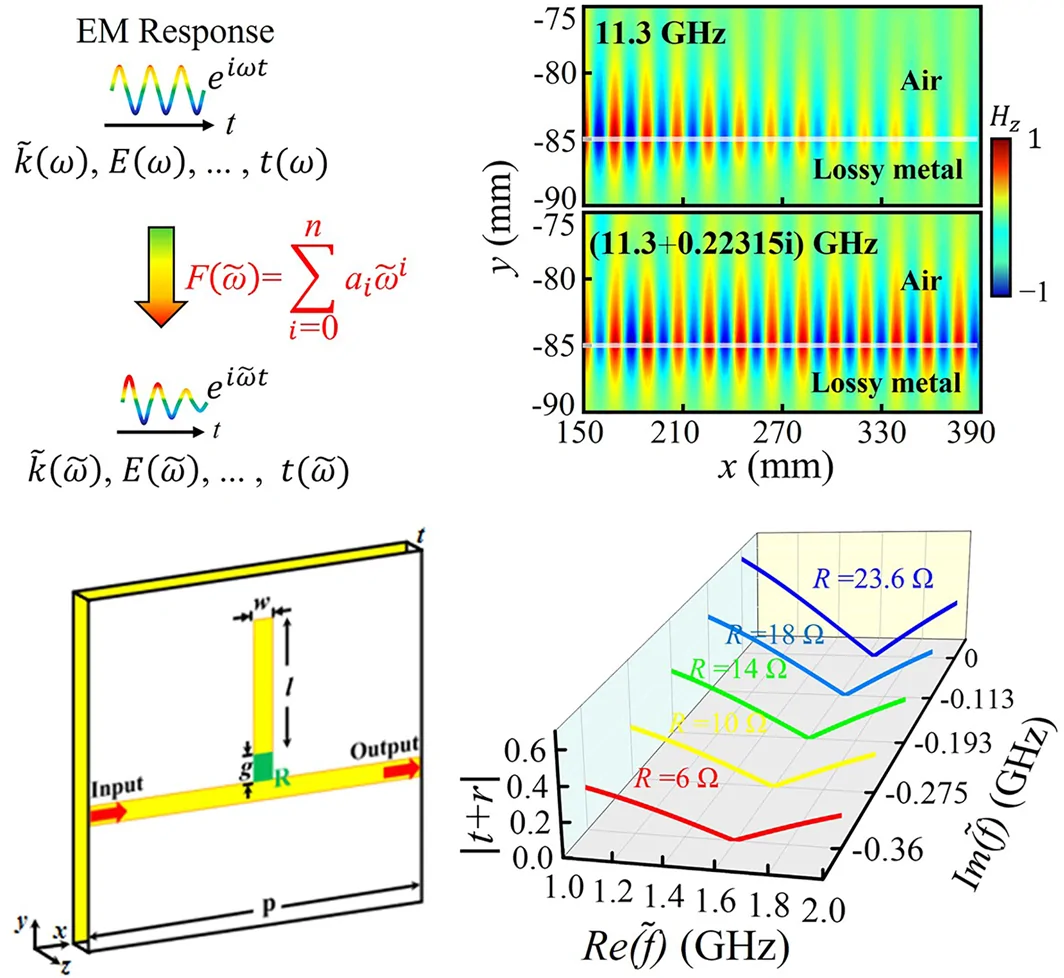

## 1 Introduction

Over recent decades, metamaterials have received great research attention owing to their excellent abilities for manipulating electromagnetic (EM) waves, where significant phenomena have been discovered, such as negative refraction [1, 2, 3], perfect tunneling [4, 5, 6], invisibility cloaking [7, 8, 9, 10, 11], and abnormal reflection/transmission [12, 13, 14]. Nevertheless, inherent material losses [15], stemming from material absorption and scattering, hinder the practical applications of metamaterials. For instance, suffering from metal loss, the actual resolution of the superlens is substantially reduced [16]. Thus, alleviating performance downgrade caused by material losses becomes an urgent task for the metamaterial community. Several strategies, including adopting low‐loss materials [17, 18] and optimizing structure geometries [19], have been developed, but remain constrained to specific scenarios. An alternative strategy is to incorporate gain material [20, 21, 22, 23, 24, 25], yet leading to undesirable effects such as instabilities and extra noises [26, 27]. Interestingly, the excitation of complex frequency waves (CFWs) with engineered temporal profiles has been theoretically proposed to recover the lost sub‐wavelength information caused by material loss without introducing noise [28, 29], indicating that the implementation of CFW offers a better solution than introducing gain. Distinct from EM wave behaviors in the real frequency domain [30, 31, 32, 33, 34, 35, 36], CFWs exhibit unique propagation characteristics [37, 38], that is, virtual loss and virtual gain [38, 39, 40], which further lead to intriguing theoretical predictions, such as coherent virtual absorption [41, 42, 43] and virtual parity–time symmetry [44]. Consequently, the experimental realization and measurement of CFWs become vital, which facilitate the exploration of their potential applications. Time‐domain manipulations of acoustic waves to achieve CFW have been demonstrated [45, 46], where the sound imaging resolution can be enhanced by CFW with optimized temporal decay [46]. However, the direct time‐domain excitation of CFWs at high frequencies, particularly in microwave and optical regimes, remains a big challenge due to hardware limitations. Recent advances to realize CFWs by synthesizing multiple real‐frequency waves have initially demonstrated the success of offsetting material loss [47, 48, 49]. Thus, developing more strategies for high‐frequency CFW excitations will favor the development of future applications.

In this study, we propose a general method for CFW excitation. As illustrated in Figure 1, the EM responses for complex frequencies, including the wavevector, electric/magnetic field, and transmission/reflection coefficient, can be obtained from real frequencies through the introduction of a mathematical bridge function F(ω), termed the virtual CFW excitation. Specifically, we demonstrate that the propagation losses of both a point source and surface plasmon polaritons in the presence of material loss are successfully offset. In addition, we have unveiled the physical mechanism of coherent virtual absorption, eventually confirmed in proof‐of‐principle microwave experiments.

**FIGURE 1:**
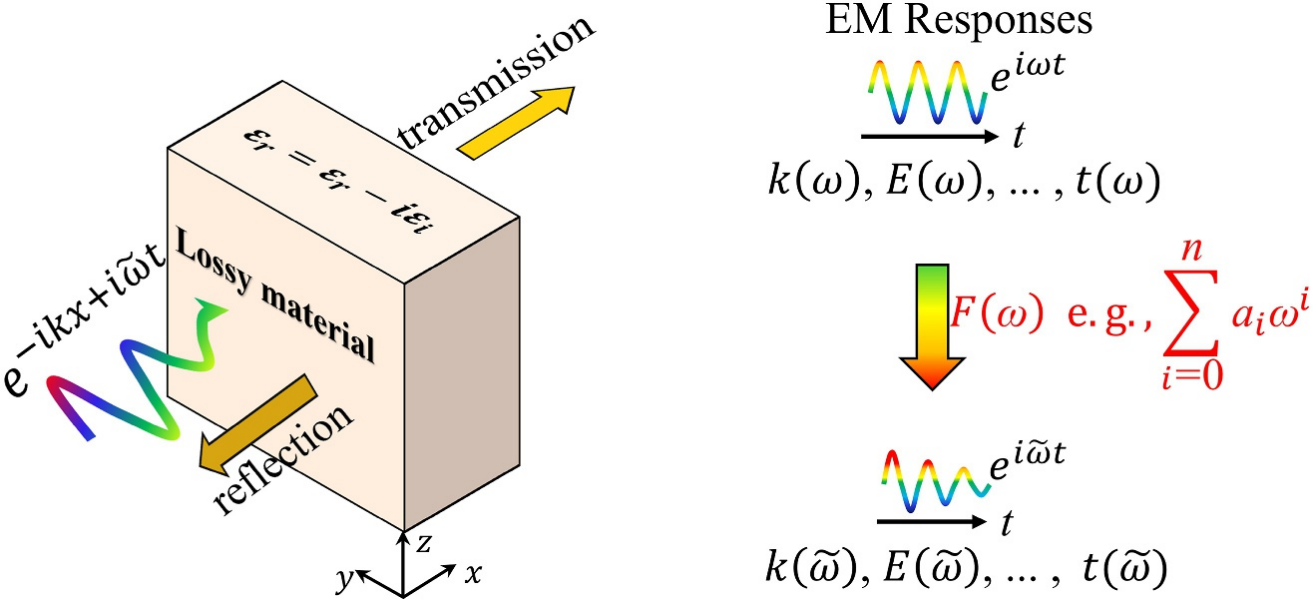
Conceptual schematic of constructing the EM responses via the virtual CFW excitation.

## 2 Results and Discussions

### 2.1 Loss Compensation for a Point Source in Lossy Media

We assume $e^{i\omega t}$ time dependence, where ω represents real angular frequency. For a conducting medium with electric conductivity σ, the corresponding complex permittivity can be given by $\tilde{\epsilon }=\epsilon_{r}-\frac{i\sigma }{\omega \;\epsilon_{0}}$ [50], where $\epsilon_{r}$ and $\epsilon_{0}$ denote the real part of relative permittivity and permittivity of free space, respectively. The complex wavevector of EM waves propagating in such a medium can be expressed as follows:

(1a) $$\tilde{k}=\frac{\omega }{c}\sqrt{\epsilon_{r}-\frac{i\sigma }{\omega \epsilon_{0}}},$$

where $\tilde{k}$ and *c* are the complex wavevector and the speed of light in free space, respectively. From Equation 1a, it is found that the non‐zero conductivity introduces a negative imaginary part to the wavevector, resulting in the propagation loss of EM waves. This physical interpretation relies on the assumption that the frequency is purely real values. If complex frequencies are considered, wavevector could become a real value, given by the following equation:

(1b) $$k=\frac{\tilde{\omega }}{c}\sqrt{\epsilon_{r}-\frac{i\sigma }{\tilde{\omega }\epsilon_{0}}},$$

where k and $\tilde{\omega }$ are the real wavevector and the complex angular frequency, respectively. Solving Equation 1b (see Supporting Information S1) enables that complex frequency $\tilde{f}=f_{r}+if_{i}$ must satisfy the following equation:

(2) $$f_{i}=\frac{\sigma \;}{{4\pi \epsilon }_{0}\epsilon_{r}},$$

here $f_{i}$ is solely determined by the ratio $\frac{\sigma }{\epsilon_{r}}$, indicating that broadband lossless propagation in lossy media is possible if a complex frequency excitation is allowed.

For simplicity, a material with $\epsilon_{r}=1$ and σ=0.1S/m is considered. By evaluating Equation 1a, we plot the real and imaginary components of the wavevector for real frequencies, as shown in Figure 2a. The results reveal the presence of a negative imaginary component in the wavevector, corresponding to the attenuation of EM waves. This attenuation can be compensated if a complex frequency with an imaginary component of 0.899GHz is excited. Calculating Equation 1b, the corresponding wavevectors under complex frequency are depicted as blue and red circles in Figure 2b, it is found that wavevectors are purely real values. To further establish a connection between real and complex frequencies, we propose a mathematical framework that enables virtual CFW excitation, allowing the wavevector at complex frequencies to be derived directly from its real‐frequency counterpart. Specifically, the dependence of the complex wavevector on frequency can be represented by a polynomial expansion

(3) $$\tilde{k}\left(f\right)=\sum_{i=0}^{n}a_{i}f^{i},$$

where n=2 and $a_{i}$ denotes the coefficient of the polynomial. The excellent agreement between analytical results, the polynomial and using the finite element method (FEM) implemented in COMSOL Multiphysics can be found in Figure 2a. By substituting real frequencies with complex frequencies in Equation (3), as shown in Figure 2b, we can retrieve the wavevectors for complex frequency, which align perfectly with the analytical expression of Equation 1b. This finding confirms that the proposed virtual CFW excitation provides an effective mathematical bridge linking real and complex frequency domains to obtain EM responses from real frequency counterparts.

**FIGURE 2:**
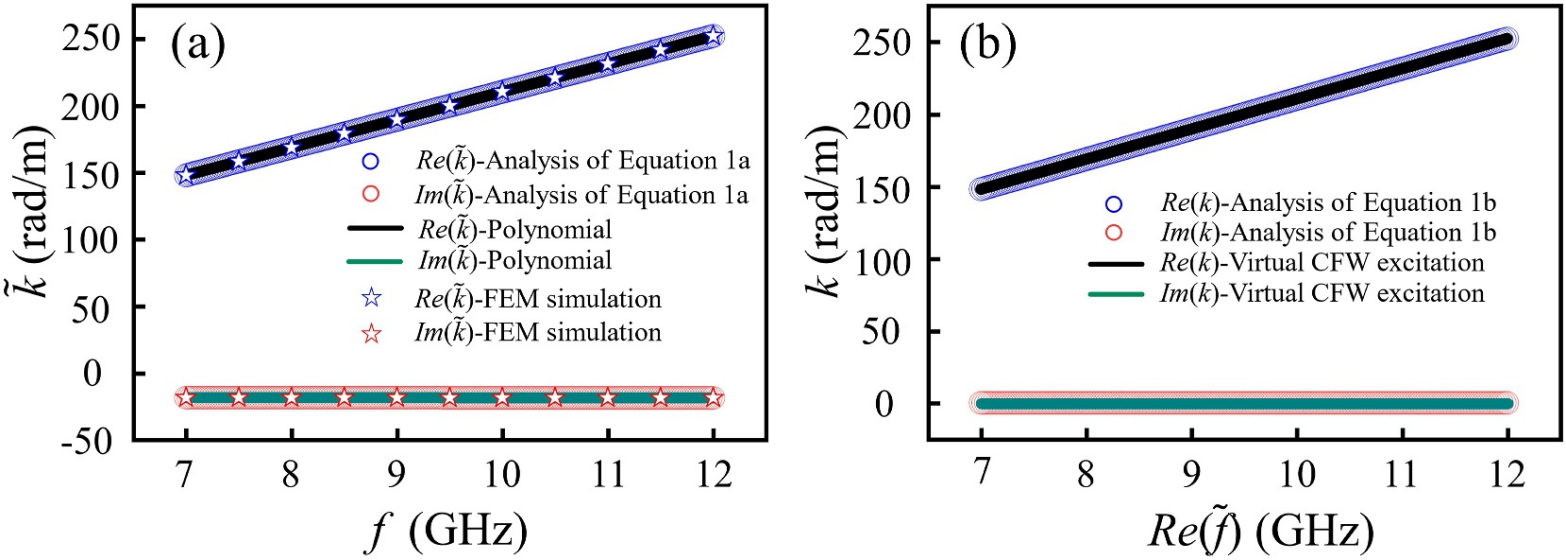
Wavevector for real and complex frequencies. (a) The calculated real and imaginary parts of the wavevector in lossy material with σ=0.1S/m for real frequencies under different approaches. (b) The wavevector in the lossy material with σ=0.1S/m for complex frequencies with a constant imaginary part $f_{i}=0.899\;\text{GHz}$ under the analysis of Equation 1b and virtual CFW excitation.

Furthermore, we proceed to demonstrate that the lost electric field information caused by material loss can be restored. Figure 3a shows a point source radiating in a lossless medium. When material loss of σ=0.1S/m is introduced, as shown in Figure 3b, the corresponding electric field $E_{z}$ decays quickly away from the source. Using the $E_{z}$‐distributions for the lossy medium over real frequency range from 10 to 12 GHz with a spacing of 0.0025 GHz, we can build a bridge function, that is, $E_{z}\left(x_{0},y_{0},f\right)=\sum_{i=0}^{n}a_{i}\left(x_{0},y_{0}\right)\;f^{i}$, where $\left(x_{0},y_{0}\right)$ refers to the spatial position. By inserting the complex frequencies with an imaginary part of 0.899GHz into the polynomial, the $E_{z}$‐distributions at different complex frequencies can be obtained, as shown in Figure 3c,d, the lost electric fields are recovered. Moreover, to identify the recovery of the electric field, we introduce the correlation coefficient. As displayed in Figure 3f, the correlation coefficient is only 85% between field distributions of a lossless medium and a lossy medium at the same real frequencies, whereas it exceeds 97.5% under virtual CFW excitation, validating the effectiveness of our proposal for field recovery. Even if a random noise is added to conductivity (0.08∼0.12S/m, while maintaining a 0.1S/m average value), a similar undistorted $E_{z}$‐distribution at complex frequency is displayed in Figure 3e with an above 95% correlation coefficient. The robustness is thus verified, and more details for random noise can be found in Supporting Information S1.

**FIGURE 3:**
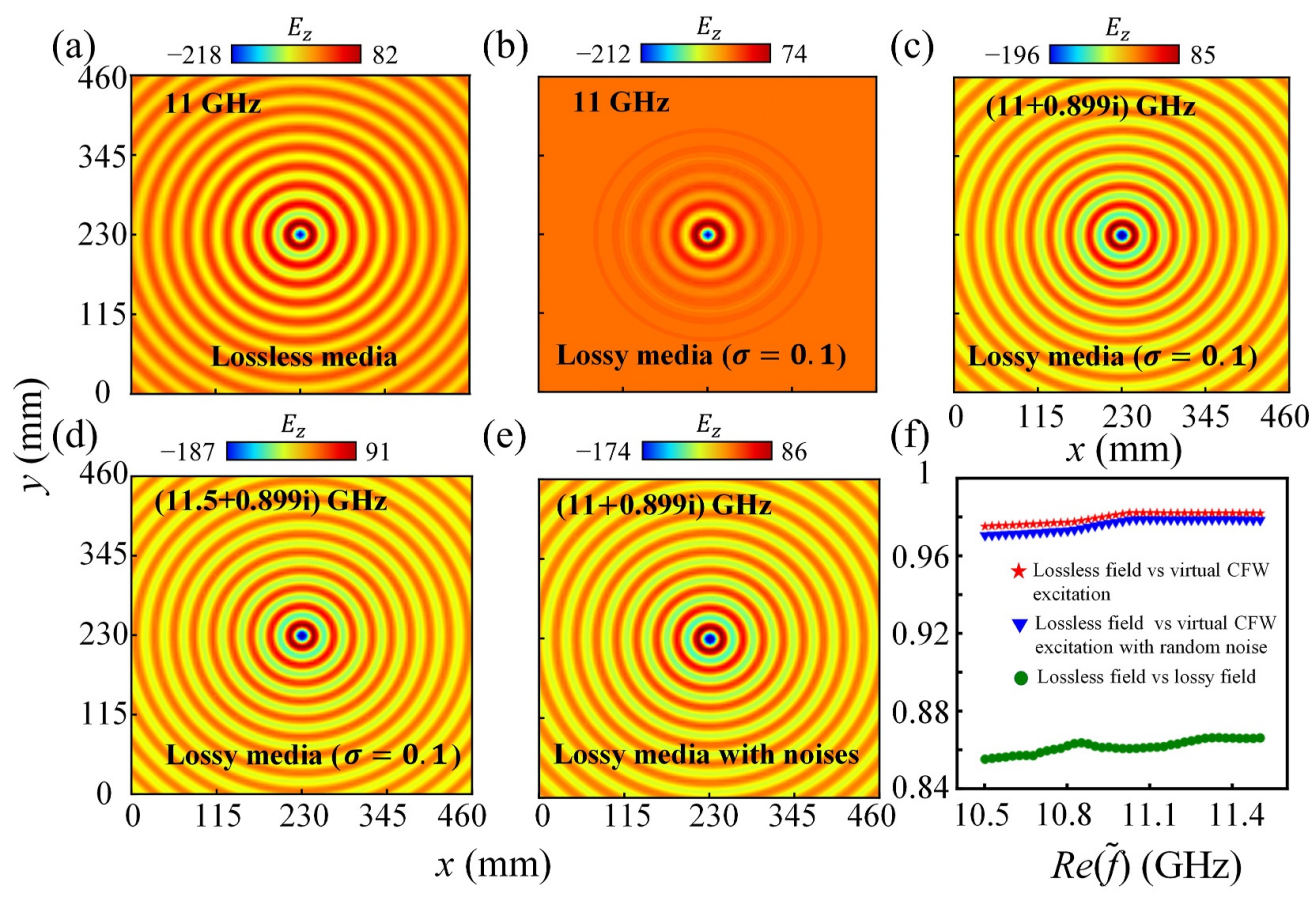
The radiation of a point source in a lossless and a lossy medium for real and complex frequency excitations. (a) and (b) The simulated $E_{z}$‐distributions inside a lossless medium and a lossy medium at working frequency f=11GHz. (c) and (d) The obtained $E_{z}$‐distributions inside a lossy medium for different complex frequencies. (e) The obtained $E_{z}$‐distributions inside a lossy medium with random noise at the working frequency $\tilde{f}=11+0.899i$ GHz. (f) The calculated correlation coefficient of the field distributions at different frequencies.

### 2.2 Loss Compensation of Surface Plasmon Polariton

The virtual CFW excitation can also be utilized to compensate for the propagation loss of surface plasmon polariton (SPP), as a demonstration to recover evanescent magnetic fields. The surface plasmon polariton, supporting only transverse electric mode with magnetic field polarized out of the plane, is confined at the interface between a dielectric and metal [51, 52, 53], exhibiting significant potentials for energy transfer [54, 55, 56, 57], and biosensors [58, 59, 60]. Here, we consider the SPPs propagate along the interface between air ($\epsilon_{d}=1$) and a lossy metal with $\epsilon_{m}=-2-\frac{i\;\sigma }{\omega \;\epsilon_{0}}$ and the corresponding dispersion relation is given by ${\tilde{k}}_{\text{spp}}=\frac{\omega }{c}\sqrt{\frac{\epsilon_{d}\;\epsilon_{m}}{\epsilon_{d}+\epsilon_{m}}}$ [52]. Because of the wavevector mismatch between SPPs and free space waves, total internal reflection [61] is used to excite the SPPs by an isosceles‐right‐triangle‐shaped prism with permittivity $\epsilon_{\text{prism}}=4$, as shown in Figure 4a. When the conductivity is set as σ=0S/m, the SPP propagates along the interface between the lossless metal and air without dissipation, as shown in Figure 4a, we highlight the corresponding $H_{z}$‐distribution within the red wireframe in Figure 4c. When lossy metal with σ=0.05S/m is considered, as shown in Figure 4b–d, $H_{z}$ quickly decays along the propagation direction. Using virtual CFW excitation, we can also construct a polynomial to depict the magnetic field for real frequencies, that is, $H_{z}\left(x_{0},y_{0},f\right)=\sum_{i=0}^{n}a_{i}\left(x_{0},y_{0}\right)\;f^{i}$. The imaginary part of complex frequency can be derived as $f_{i}\approx 0.22315\;\text{GHz}$ by solving $\text{Im}\left({\tilde{k}}_{\text{spp}}\right)=0$, implying that when a complex frequency wave is excited, the propagation loss can be eliminated. Employing virtual CFW excitation by inserting complex frequencies into polynomial, as shown in Figure 4e,f, lossless propagation of SPPs can be observed along the interface for different complex frequencies. More investigations for SPPs at an interface between air and dispersive metal can be found in Supporting Information S1.

**FIGURE 4:**
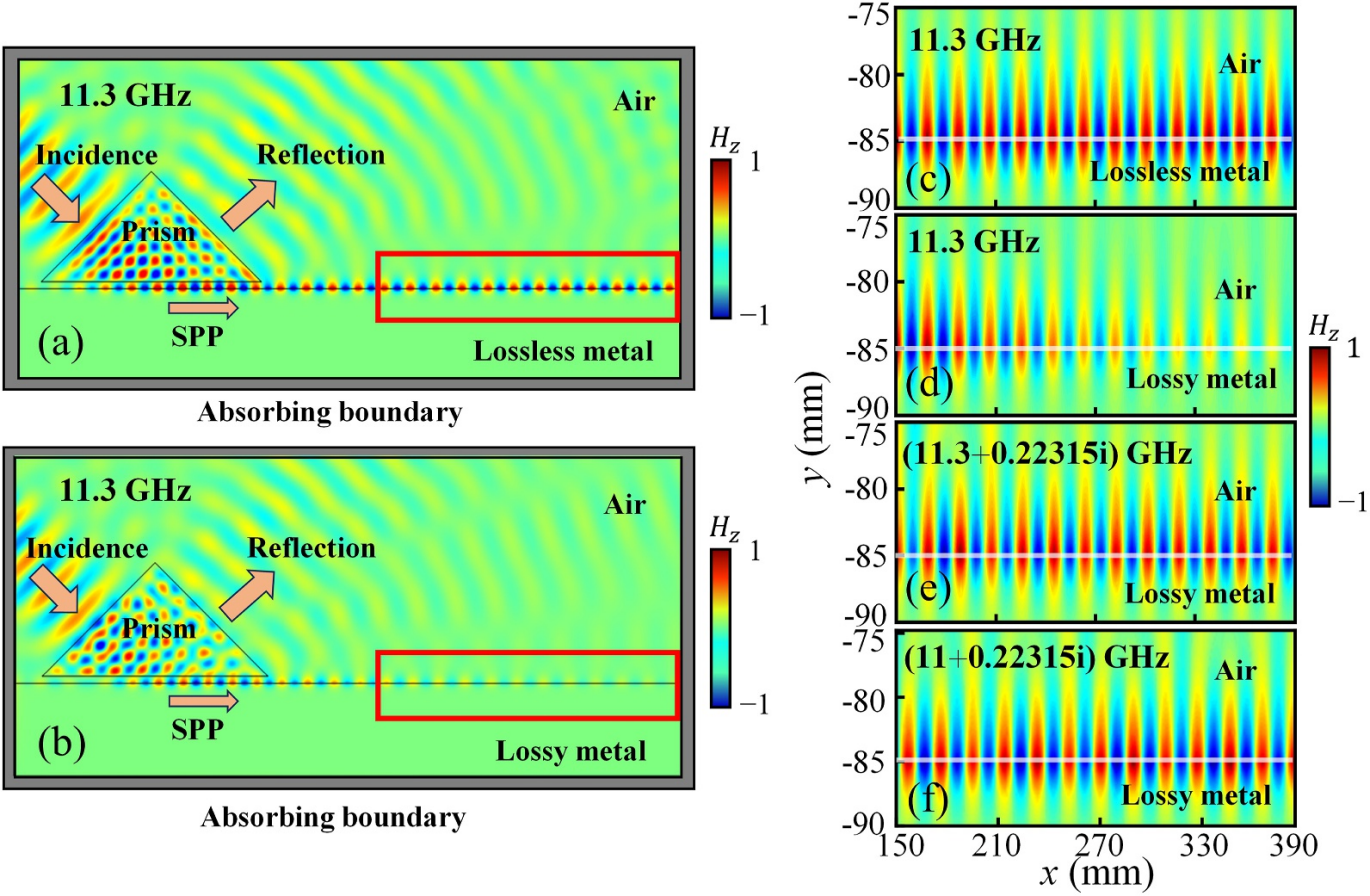
The $H_{z}$‐distributions of the SPPs for real and complex frequency excitations. (a) The simulated SPP propagation between air and lossless metal at f=11.3GHz. (b) The simulated SPP propagation between air and lossy metal at f=11.3GHz. The pink arrows indicate the propagation direction of waves. The right triangle region is the prism for evanescent excitation. (c) and (d) The highlighted $H_{z}$‐distributions in (a), in (b). (e) and (f) The $H_{z}$‐distributions at $\tilde{f}=11.3+0.22315i$GHz and $\tilde{f}=11+0.22315i$GHz, respectively. The white line denotes the interface between air and metal.

### 2.3 Experimental Realization of Coherent Virtual Absorption

Transmission and reflection are also important characteristics for EM waves, which are associated with EM absorption. A typical example of complex frequency is coherent virtual absorption [41, 42], whereas its experimental realization is still a big challenge due to the lack of experimental techniques for complex frequency excitations in microwave and optical regimes. Here, using virtual CFW excitation, coherent virtual absorption is experimentally realized through a microwave transmission line, which is, to the best of our knowledge, the first experimental verification in optics. The transmission line is displayed in Figure 5a, composed of the copper microstrip line and patch printed on FR‐4 substrate (relative permittivity $\epsilon_{r}=4.5$) with the metallic reflector. A resistor is integrated into the gap between the microstrip line and the metallic patch, where the geometry parameters are *p* = 50 mm, *l* = 20 mm, *g* = 1 mm, and *w* = 2.8 mm, and the thickness of the FR‐4 substrate is *t* = 1.5 mm. The coupled mode theory (CMT) [62, 63, 64] analysis of such a transmission line can be found in Supporting Information S1.

**FIGURE 5:**
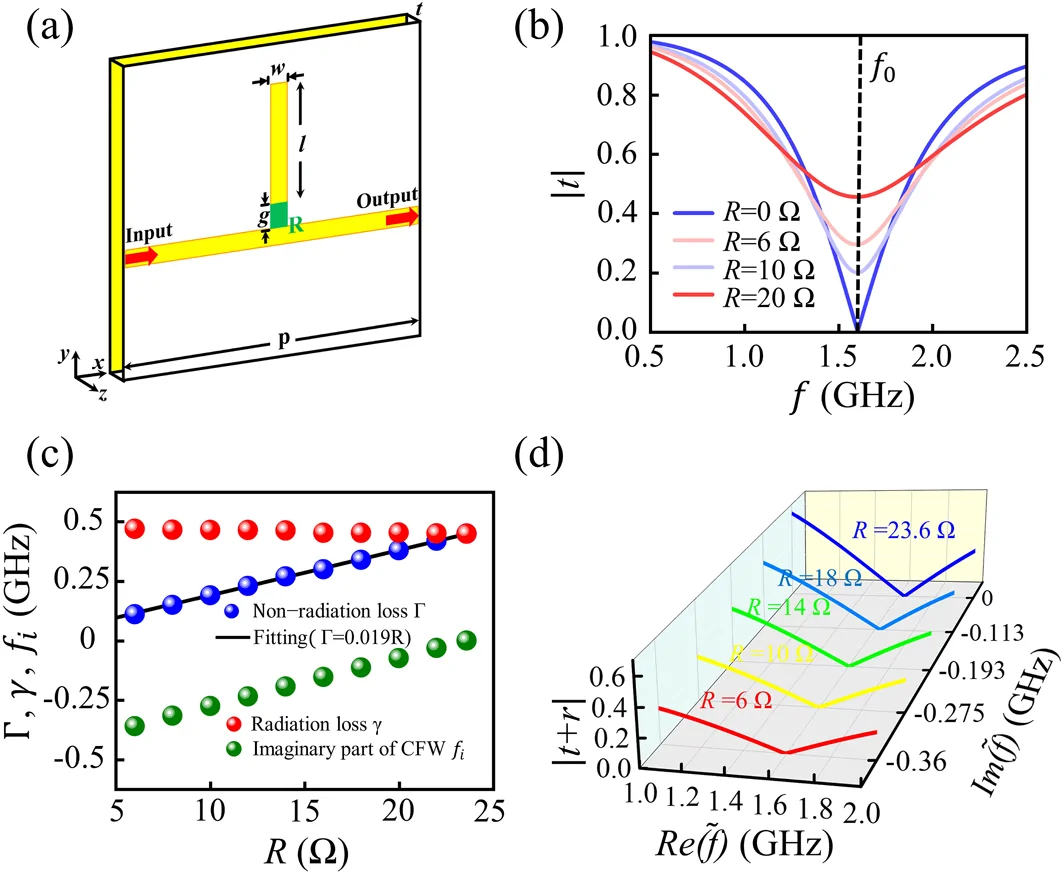
Simulations for the coherent virtual absorption. (a) The schematic illustration of the transmission line. (b) The simulated transmission coefficients at different resistors. (c) Retrieved non‐radiation loss, radiation loss and the corresponding imaginary part of complex frequencies at different resistors integrated. (d) |t+r| for different resistors in the complex frequency plane.

Simulated by the finite difference time domain method (CST Microwave Studio), the transmission coefficients for different resistors are displayed in Figure 5b, from which the resonant frequency is found at $f_{0}=1.6\;\text{GHz}$, whereas the non‐radiation loss Γ can be controlled by tuning the integrated resistors. As shown in Figure 5c, it is found that non‐radiation loss Γ is a linear dependence on the resistance of the integrated resistor R as Γ=0.019R, where the radiation loss γ is nearly a constant (∼0.45 GHz). Two counter‐propagating CFWs are required to induce coherent virtual absorption with t+r=0, whose frequency should satisfy $\tilde{f}=f_{0}-i\left(\gamma -\Gamma \right)$ (see Supporting Information S1). Similarly, we can also build a polynomial to describe the dependence of t+r on real frequencies, that is, $t+r=\sum_{i=0}^{n}a_{i}f^{i}$. By introducing the complex frequency $\tilde{f}$ into the polynomial, we can obtain |t+r| as a function of complex frequencies for different resistors, as shown in Figure 5d, |t+r| reach zero at the desired complex frequencies, indicating that coherent virtual absorption occurs. It is worth noting that for some electromagnetic responses with strong dispersions, the polynomial fitting may fail and other fitting functions, that is, rational fitting or Lorentzian fitting, shall be considered, more details can be found in Supporting Information S1.

Finally, coherent virtual absorption is visualized by our proof‐of‐principle experiments. As depicted in Figure 6a, the two ports of the experimental sample are connected to the vector network analyzer (Keysight E5071C) for transmission/reflection coefficient measurements. Different resistances, that is, R=8.6Ω,10.2Ω,12.5Ω,16.6Ω,22.6Ω, can be integrated into the transmission line. By implementing virtual CFW excitation for measured transmission and reflection coefficients, we can obtain |t+r| as a function of the real and imaginary parts of the complex frequency, as shown in Figure 6b, for R=12.5Ω, coherent virtual absorption occurs at $\tilde{f}=1.665-0.194i\;\text{GHz}$ with a minimal value of |t+r|. Here, we note that the real part of the frequency deviates slightly from the theoretical prediction due to the imperfect fabrication of the sample. Moreover, |t+r| for R=12.5Ω is plotted in a green line of Figure 6c, and similar spectra can be found for other integrated resistances, indicating the success of demonstrating coherent virtual absorption. Figure 6d displays the experimentally extracted radiation loss, non‐radiation loss, and imaginary part of complex frequency as a function of resistances, in accord with our theoretical prediction. The effects of amplitude and phase imbalance between two inputs can be found in Supporting Information S1.

**FIGURE 6:**
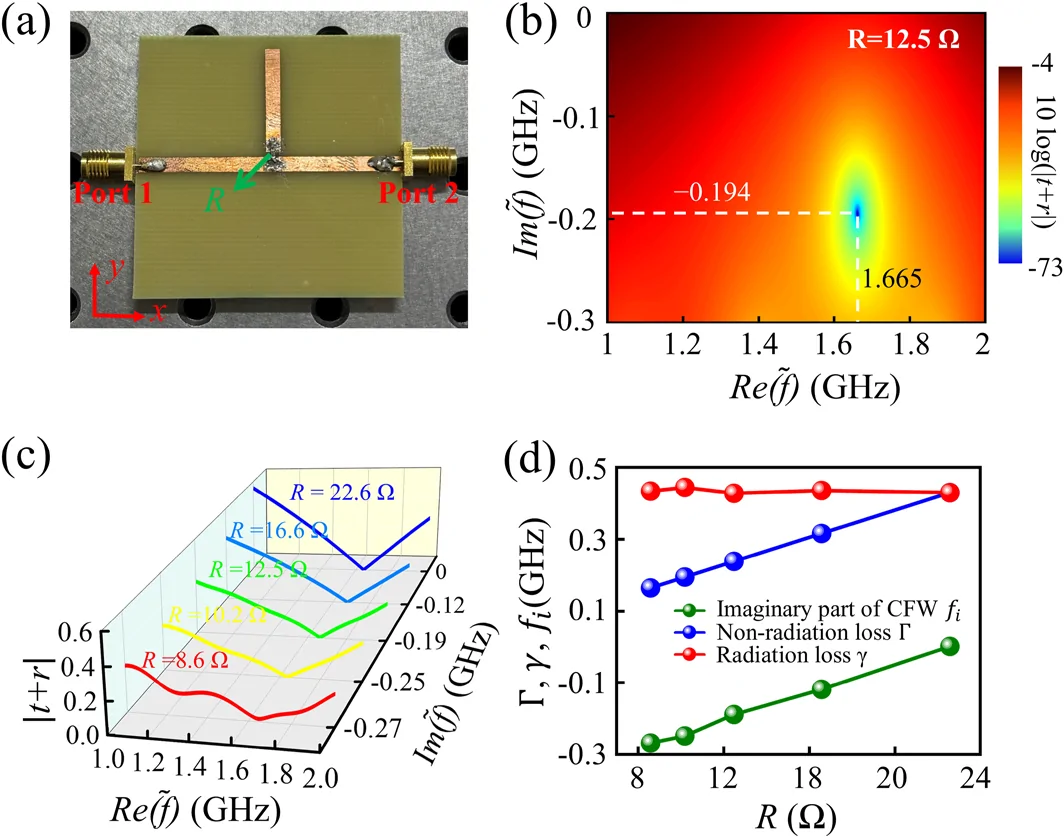
The experimental realization for coherent virtual absorption. (a) The photograph of the experimental sample. (b) The |t+r| as a function of the real and imaginary parts of complex frequencies for an integrated resistor R=12.5Ω. (c) Experimentally measured |t+r| for different resistors. (d) Experimentally retrieved radiation loss, non‐radiation loss, and imaginary part of frequency as a function of resistance.

## 3 Conclusions

In summary, we have successfully proposed a virtual CFW excitation to study the properties of CFWs. Through constructing a mathematical bridge between real and complex frequencies, any EM responses for complex frequencies can be obtained from the real frequency domain. We demonstrate that CFWs can compensate for the propagation loss of both point sources and the SPPs even in the presence of noise. Finally, the coherent virtual absorption is experimentally demonstrated. Although our work is only verified in microwave experiments, the method is general and can also be extended to the optical regime, which will open up more opportunities to investigate wave physics in the complex frequency domain.

## Author Contributions

Sheng Zhang: conceptualization, investigation, validation, software, formal analysis, visualization, writing – review and editing, writing – original draft. Zhi Hong Hang: conceptualization, supervision, funding acquisition, writing – review and editing, writing – original draft, project administration, resources.

## Conflicts of Interest

The authors declare no conflicts of interest.

## Supporting information


Supporting Information S1

## Data Availability

Data underlying the results presented in this paper are not publicly available at this time but may be obtained from the authors upon reasonable request.

## References

[1] J. B. Pendry , “Negative Refraction Makes a Perfect Lens,” Physical Review Letters 85, no. 18 (2000): 3966–3969, 10.1103/physrevlett.85.3966. 11041972

[2] R. A. Shelby , D. R. Smith , and S. Schultz , “Experimental Verification of a Negative Index of Refraction,” Science 292, no. 5514 (2001): 77–79, 10.1126/science.1058847. 11292865

[3] D. R. Smith , J. B. Pendry , and M. C. Wiltshire , “Metamaterials and Negative Refractive Index,” Science 305, no. 5685 (2004): 788–792, 10.1126/science.1096796. 15297655

[4] R. Liu , Q. Cheng , T. Hand , et al., “Experimental Demonstration of Electromagnetic Tunneling Through an Epsilon‐Near‐Zero Metamaterial at Microwave Frequencies,” Physical Review Letters 100, no. 2 (2008): 023903, 10.1103/physrevlett.100.023903. 18232869

[5] M. Silveirinha and N. Engheta , “Tunneling of Electromagnetic Energy Through Subwavelength Channels and Bends Using ε‐Near‐Zero Materials,” Physical Review Letters 97, no. 2006 (2006): 157403, 10.1103/physrevlett.97.157403. 17155357

[6] X. Huang , Y. Lai , Z. H. Hang , H. Zheng , and C. T. Chan , “Dirac Cones Induced by Accidental Degeneracy in Photonic Crystals and Zero‐Refractive‐Index Materials,” Nature Materials 10, no. 8 (2011): 582–586, 10.1038/nmat3030. 21623377

[7] D. Schurig , J. J. Mock , B. Justice , et al., “Metamaterial Electromagnetic Cloak at Microwave Frequencies,” Science 314, no. 5801 (2006): 977–980, 10.1126/science.1133628. 17053110

[8] J. B. Pendry , D. Schurig , and D. R. Smith , “Controlling Electromagnetic Fields,” Science 312, no. 5781 (2006): 1780–1782, 10.1126/science.1125907. 16728597

[9] W. Cai , U. K. Chettiar , A. V. Kildishev , and V. M. Shalaev , “Optical Cloaking With Metamaterials,” Nature Photonics 1, no. 4 (2007): 224–227, 10.1038/nphoton.2007.28.

[10] H. Chu , Q. Li , B. Liu , et al., “A Hybrid Invisibility Cloak Based on Integration of Transparent Metasurfaces and Zero‐Index Materials,” Light: Science & Applications 7, no. 1 (2018): 50, 10.1038/s41377-018-0052-7. PMC6107001 30839599

[11] Y. Lai , H. Chen , Z.‐Q. Zhang , and C. T. Chan , “Complementary Media Invisibility Cloak That Cloaks Objects at a Distance Outside the Cloaking Shell,” Physical Review Letters 102, no. 9 (2009): 093901, 10.1103/physrevlett.102.093901. 19392518

[12] N. Yu , P. Genevet , M. A. Kats , et al., “Light Propagation With Phase Discontinuities: Generalized Laws of Reflection and Refraction,” Science 334, no. 6054 (2011): 333–337, 10.1126/science.1210713. 21885733

[13] S. Sun , Q. He , S. Xiao , Q. Xu , X. Li , and L. Zhou , “Gradient‐Index Meta‐Surfaces as a Bridge Linking Propagating Waves and Surface Waves,” Nature Materials 11, no. 5 (2012): 426–431, 10.1038/nmat3292. 22466746

[14] C. Pfeiffer and A. Grbic , “Metamaterial Huygens’ Surfaces: Tailoring Wave Fronts With Reflectionless Sheets,” Physical Review Letters 110, no. 19 (2013): 197401, 10.1103/physrevlett.110.197401. 23705738

[15] J. B. Khurgin , “How to Deal With the Loss in Plasmonics and Metamaterials,” Nature Nanotechnology 10, no. 1 (2015): 2–6, 10.1038/nnano.2014.310. 25559961

[16] N. Fang , H. Lee , C. Sun , and X. Zhang , “Sub‐Diffraction‐Limited Optical Imaging With a Silver Superlens,” Science 308, no. 5721 (2005): 534–537, 10.1126/science.1108759. 15845849

[17] A. Boltasseva and H. A. Atwater , “Low‐Loss Plasmonic Metamaterials,” Science 331, no. 6015 (2011): 290–291, 10.1126/science.1198258. 21252335

[18] B.‐I. Popa and S. A. Cummer , “Compact Dielectric Particles as a Building Block for Low‐Loss Magnetic Metamaterials,” Physical Review Letters 100, no. 20 (2008): 207401, 10.1103/physrevlett.100.207401. 18518576

[19] D. Ö. Güney , T. Koschny , and C. M. Soukoulis , “Reducing Ohmic Losses in Metamaterials by Geometric Tailoring,” Physical Review B: Condensed Matter and Materials Physics 80, no. 12 (2009): 125129, 10.1103/physrevb.80.125129.

[20] S. A. Ramakrishna and J. B. Pendry , “Removal of Absorption and Increase in Resolution in a Near‐Field Lens via Optical Gain,” Physical Review B 67, no. 20 (2003): 201101, 10.1103/physrevb.67.201101.

[21] S. Xiao , V. P. Drachev , A. V. Kildishev , et al., “Loss‐Free and Active Optical Negative‐Index Metamaterials,” Nature 466, no. 7307 (2010): 735–738, 10.1038/nature09278. 20686570

[22] S. Wuestner , A. Pusch , K. L. Tsakmakidis , J. M. Hamm , and O. Hess , “Overcoming Losses With Gain in a Negative Refractive Index Metamaterial,” Physical Review Letters 105, no. 12 (2010): 127401, 10.1103/physrevlett.105.127401. 20867669

[23] J. Grgić , J. R. Ott , F. Wang , et al., “Fundamental Limitations to Gain Enhancement in Periodic Media and Waveguides,” Physical Review Letters 108, no. 18 (2012): 183903, 10.1103/physrevlett.108.183903. 22681078

[24] D. Ye , K. Chang , L. Ran , and H. Xin , “Microwave Gain Medium With Negative Refractive Index,” Nature Communications 5, no. 1 (2014): 5841, 10.1038/ncomms6841. 25524752

[25] M. Sadatgol , Ş.K. Özdemir , L. Yang , and D. Ö. Güney , “Plasmon Injection to Compensate and Control Losses in Negative Index Metamaterials,” Physical Review Letters 115, no. 3 (2015): 035502, 10.1103/physrevlett.115.035502. 26230802

[26] A. D. Boardman , V. V. Grimalsky , Y. S. Kivshar , et al., “Active and Tunable Metamaterials,” Laser & Photonics Reviews 5, no. 2 (2010): 287–307, 10.1002/lpor.201000012.

[27] M. I. Stockman , “Spaser Action, Loss Compensation, and Stability in Plasmonic Systems With Gain,” Physical Review Letters 106, no. 15 (2011): 156802, 10.1103/physrevlett.106.156802. 21568593

[28] A. Archambault , M. Besbes , and J.‐J. Greffet , “Superlens in the Time Domain,” Physical Review Letters 109, no. 9 (2012): 097405, 10.1103/physrevlett.109.097405. 23002884

[29] S. Kim , A. Krasnok , and A. Alù , “Complex‐Frequency Excitations in Photonics and Wave Physics,” Science 387, no. 6741 (2025): eado4128, 10.1126/science.ado4128. 40146845

[30] Y. D. Chong , L. Ge , H. Cao , and A. D. Stone , “Coherent Perfect Absorbers: Time‐Reversed Lasers,” Physical Review Letters 105, no. 5 (2010): 053901, 10.1103/physrevlett.105.053901. 20867918

[31] R. Fleury , D. L. Sounas , and A. Alù , “Negative Refraction and Planar Focusing Based on Parity‐Time Symmetric Metasurfaces,” Physical Review Letters 113, no. 2 (2014): 023903, 10.1103/physrevlett.113.023903. 25062184

[32] S. Li , J. Luo , S. Anwar , et al., “Broadband Perfect Absorption of Ultrathin Conductive Films With Coherent Illumination: Superabsorption of Microwave Radiation,” Physical Review B 91, no. 22 (2015): 220301(R), 10.1103/physrevb.91.220301.

[33] D. L. Sounas , R. Fleury , and A. Alù , “Unidirectional Cloaking Based on Metasurfaces With Balanced Loss and Gain,” Physical Review Applied 4, no. 1 (2015): 014005, 10.1103/physrevapplied.4.014005.

[34] R. El‐Ganainy , K. G. Makris , M. Khajavikhan , Z. H. Musslimani , S. Rotter , and D. N. Christodoulides , “Non‐Hermitian Physics and PT Symmetry,” Nature Physics 14, no. 1 (2018): 11–19, 10.1038/nphys4323.

[35] J. Luo , J. Li , and Y. Lai , “Electromagnetic Impurity‐immunity Induced by Parity‐Time Symmetry,” Physical Review X 8, no. 3 (2018): 031035, 10.1103/physrevx.8.031035.

[36] J. Luo , B. Liu , Z. H. Hang , and Y. Lai , “Coherent Perfect Absorption via Photonic Doping of Zero‐Index Media,” Laser & Photonics Reviews 12, no. 8 (2018): 1800001, 10.1002/lpor.201800001.

[37] S. An , T. Liu , J. Zhu , and L. Cheng , “Complex‐Frequency Calculation in Acoustics With Real‐Frequency Solvers,” Physical Review B 111, no. 2 (2025): L020301, 10.1103/physrevb.111.l020301.

[38] G. P. Zouros , I. Loulas , E. Almpanis , A. Krasnok , and K. L. Tsakmakidis , “Anisotropic Virtual Gain and Large Tuning of Particles’ Scattering by Complex‐Frequency Excitations,” Communications Physics 7, no. 1 (2024): 283, 10.1038/s42005-024-01772-w.

[39] K. L. Tsakmakidis , T. W. Pickering , J. M. Hamm , A. F. Page , and O. Hess , “Completely Stopped and Dispersionless Light in Plasmonic Waveguides,” Physical Review Letters 112, no. 16 (2014): 167401, 10.1103/physrevlett.112.167401. 24815668

[40] A. Krasnok , D. Baranov , H. Li , M.‐A. Miri , F. Monticone , and A. Alú , “Anomalies in Light Scattering,” Advances in Optics and Photonics 11, no. 4 (2019): 892, 10.1364/aop.11.000892.

[41] D. G. Baranov , A. Krasnok , and A. Alù , “Coherent Virtual Absorption Based on Complex Zero Excitation for Ideal Light Capturing,” Optica 4, no. 12 (2017): 1457, 10.1364/optica.4.001457.

[42] Q. Zhong , L. Simonson , T. Kottos , and R. El‐Ganainy , “Coherent Virtual Absorption of Light in Microring Resonators,” Physical Review Research 2, no. 1 (2020): 013362, 10.1103/physrevresearch.2.013362.

[43] I. Loulas , E. C. Psychogiou , K. L. Tsakmakidis , and N. Stefanou , “Analytic Theory of Complex‐Frequency‐Aided Virtual Absorption,” Optics Express 33, no. 13 (2025): 28333–28342, 10.1364/oe.563570. 40798355

[44] H. Li , A. Mekawy , A. Krasnok , and A. Alù , “Virtual Parity‐Time Symmetry,” Physical Review Letters 124, no. 19 (2020): 193901, 10.1103/physrevlett.124.193901. 32469571

[45] Z. Gu , H. Gao , H. Xue , J. Li , Z. Su , and J. Zhu , “Transient Non‐Hermitian Skin Effect,” Nature Communication 13, no. 1 (2022): 7668, 10.1038/s41467-022-35448-2. PMC9744917 36509774

[46] A. Ghoshroy , Ş.K. Özdemir , and D. Ö. Güney , “Loss Compensation in Metamaterials and Plasmonics With Virtual Gain,” Optical Materials Express 10, no. 8 (2020): 1862, 10.1364/ome.397720.

[47] F. Guan , X. Guo , K. Zeng , et al., “Overcoming Losses in Superlenses With Synthetic Waves of Complex Frequency,” Science 381, no. 6659 (2023): 766–771, 10.1126/science.adi1267. 37590345

[48] F. Guan , X. Guo , S. Zhang , et al., “Compensating Losses in Polariton Propagation With Synthesized Complex Frequency Excitation,” Nature Materials 23, no. 4 (2024): 506–511, 10.1038/s41563-023-01787-8. 38191633

[49] F. Guan , Z. Lin , S. Chen , X. Wen , T. Li , and S. Zhang , “High‐Order Virtual Gain for Optical Loss Compensation in Plasmonic Metamaterials,” Nature Physics 22, no. 3 (2026): 489–494, 10.1038/s41567-026-03171-0.

[50] D. J. Griffiths , Introduction to Electrodynamics (Cambridge University Press, 2023).

[51] W. L. Barnes , A. Dereux , and T. W. Ebbesen , “Surface Plasmon Subwavelength Optics,” Nature 424, no. 6950 (2003): 824–830, 10.1038/nature01937. 12917696

[52] W. X. Tang , H. C. Zhang , H. F. Ma , W. X. Jiang , and T. J. Cui , “Concept, Theory, Design, and Applications of Spoof Surface Plasmon Polaritons at Microwave Frequencies,” Advanced Optical Materials 7, no. 1 (2018): 1800421, 10.1002/adom.201800421.

[53] F. J. Garcia‐Vidal , A. I. Fernández‐Domínguez , L. Martin‐Moreno , et al., “Spoof Surface Plasmon Photonics,” Reviews of Modern Physics 94, no. 2 (2022): 025004, 10.1103/revmodphys.94.025004.

[54] J.‐C. Weeber , A. Dereux , C. Girard , J. R. Krenn , and J.‐P. Goudonnet , “Plasmon Polaritons of Metallic Nanowires for Controlling Submicron Propagation of Light,” Physical Review B 60, no. 12 (1999): 9061–9068, 10.1103/physrevb.60.9061.

[55] H. F. Ma , X. Shen , Q. Cheng , W. X. Jiang , and T. J. Cui , “Broadband and High‐Efficiency Conversion From Guided Waves to Spoof Surface Plasmon Polaritons,” Laser & Photonics Reviews 8, no. 1 (2014): 146–151, 10.1002/lpor.201300118.

[56] Y. Meng , H. Ma , J. Wang , Y. Li , Z. Li , and S. Qu , “Broadband Spoof Surface Plasmon Polaritons Coupler Based on Dispersion Engineering of Metamaterials,” Applied Physics Letters 111, no. 15 (2017): 151904, 10.1063/1.4995505.

[57] X. Shen and T. J. Cui , “Ultrathin Plasmonic Metamaterial for Spoof Localized Surface Plasmons,” Laser & Photonics Reviews 8, no. 1 (2014): 137–145, 10.1002/lpor.201300144.

[58] A. V. Zayats , I. I. Smolyaninov , and A. A. Maradudin , “Nano‐Optics of Surface Plasmon Polaritons,” Physics Reports 408, no. 3–4 (2005): 131–314, 10.1016/j.physrep.2004.11.001.

[59] O. Salihoglu , S. Balci , and C. Kocabas , “Plasmon‐Polaritons on Graphene‐Metal Surface and Their Use in Biosensors,” Applied Physics Letters 100, no. 21 (2012): 213110, 10.1063/1.4721453.

[60] X. Zhang , W. Y. Cui , Y. Lei , X. Zheng , J. Zhang , and T. J. Cui , “Spoof Localized Surface Plasmons for Sensing Applications,” Advanced Materials Technologies 6, no. 4 (2021): 2000863, 10.1002/admt.202000863.

[61] A. Otto , “Excitation of Nonradiative Surface Plasma Waves in Silver by the Method of Frustrated Total Reflection,” Zeitschrift für Physik A Hadrons and nuclei 216, no. 4 (1968): 398–410, 10.1007/bf01391532.

[62] Y. Sun , W. Tan , H.‐Q. Li , J. Li , and H. Chen , “Experimental Demonstration of a Coherent Perfect Absorber With PT Phase Transition,” Physical Review Letters 112, no. 14 (2014): 143903, 10.1103/physrevlett.112.143903. 24765965

[63] Y.‐P. Wang , J. W. Rao , Y. Yang , et al., “Nonreciprocity and Unidirectional Invisibility in Cavity Magnonics,” Physical Review Letters 123, no. 12 (2019): 127202, 10.1103/physrevlett.123.127202. 31633946

[64] J. W. Rao , Y. T. Zhao , Y. S. Gui , X. L. Fan , D. S. Xue , and C.‐M. Hu , “Controlling Microwaves in Non‐Hermitian Metamaterials,” Physical Review Applied 15, no. 2 (2021): L021003, 10.1103/physrevapplied.15.l021003.

