# Supplementary material for: Loss Compensation and Coherent Virtual Absorption via Virtual Complex Frequency Wave Excitation
Source: Nanophotonics. 2026 May 8;15(9):e70125. doi: 10.1002/nap2.70125 (PMC13642498; doi:10.1002/nap2.70125)
Supplement: Supplementary file 1 [file NAP2-15-e70125-s001.docx]

**Supporting Information**

**Loss compensation and coherent virtual absorption via virtual complex frequency wave excitation**

Sheng Zhang, Zhi Hong Hang

School of Physical Science and Technology & Institute for Advanced Study

Soochow University, Suzhou 215006, P. R. China

E-mail: [zhhang@suda.edu.cn](mailto:zhhang@suda.edu.cn)

**1. Detailed derivation of Equation 2 in the main text**

**2. Random distribution of conductivity in lossy media**

**3. Loss compensation for SPPs at an interface between air and the Drude model metal.**

**4. The theoretical model of the coherent virtual absorption**

**5. Comparison between polynomial fitting and rational fitting**

**1. Detailed derivation of Equation 2**

The complex permittivity of lossy media with conductivity $\sigma$ is written as $\tilde{\epsilon}=\epsilon_{r}-i\frac{\sigma}{\tilde{\omega}\epsilon_{0}}$, where $-i$ denotes the material loss. In general, wavevector $\tilde{k}$and angular frequency $\tilde{\omega}$ are complex values, and the dispersion relation can be expressed as $\tilde{k}=\tilde{\omega}\sqrt{\mu_{0}\epsilon_{0}}\sqrt{\epsilon_{r}-i\frac{\sigma}{\tilde{\omega}\epsilon_{0}}}$, where $\mu_{0}$ and $\epsilon_{0}$ correspond to the permeability and permittivity of free space, respectively, the speed of light in free space is defined as $c=1/\sqrt{\mu_{0}\epsilon_{0}}$. Further, the wavevector can be written as

$\tilde{k}^{2}=\frac{\tilde{\omega}^{2}}{c^{2}}\left( \epsilon_{r}-i\frac{\sigma}{\tilde{\omega}\epsilon_{0}} \right)$,	(A1)

when the complex frequency $\tilde{\omega}=\omega_{r}+i\omega_{i}$ is incorporated into Equation A1, which can be expressed as,

$\tilde{k}^{2}c^{2}=\left( \omega_{r}^{2}+2i\omega_{r}\omega_{i}-\omega_{i}^{2} \right)\left( \epsilon_{r}-i\frac{\sigma}{\left( \omega_{r}+i\omega_{i} \right)\epsilon_{0}} \right)$,	(A2)

From Equation A2, the real and imaginary parts of $\tilde{k}^{2}c^{2}$ are respectively given by

$Re\left( \tilde{k}^{2}c^{2} \right)=\left( \omega_{r}^{2}-\omega_{i}^{2} \right)\left( \epsilon_{r}-\frac{\sigma\omega_{i}}{\left( \omega_{r}^{2}+\omega_{i}^{2} \right)\epsilon_{0}} \right)+\frac{2\omega_{i}\omega_{r}^{2}\sigma}{\left( \omega_{r}^{2}+\omega_{i}^{2} \right)\epsilon_{0}}$,	(A3)

$Im\left( \tilde{k}^{2}c^{2} \right)={-(\omega}_{r}^{2}-\omega_{i}^{2})\frac{\sigma\omega_{r}}{\left( \omega_{r}^{2}+\omega_{i}^{2} \right)\epsilon_{0}}+2\omega_{i}\omega_{r}(\epsilon_{r}-\frac{\sigma\omega_{i}}{\left( \omega_{r}^{2}+\omega_{i}^{2} \right)\epsilon_{0}})$,	(A4)

As $\omega_{r}>0$, the condition for lossless propagation requires $Im\left( \tilde{k}^{2}c^{2} \right)=0$, which is expressed as

$\omega_{i}=\frac{\sigma}{{2\epsilon}_{0}\epsilon_{r}}$,	(A5)

**2. Random distribution of conductivity in lossy media**

The arrangement of conductivity $\sigma$ is from 0.08 S/m to 0.12 S/m, and an average value is set as 0.1 S/m. The detailed distribution is shown in **Figure A1**a, where the corresponding $\text{E}_{\text{z}}$-distribution at 11 GHz is shown in Figure A1b, where the $\text{E}_{\text{z}}$-field decays quickly away from the source. Using virtual CFW excitation, we can restore the $\text{E}_{\text{z}}$-distribution and compensate for propagation loss, as shown in Figure A1c (identical to Figure 3(e) in the main text). Besides, we further increase the conductivity randomness from 0.07 S/m to 0.13 S/m, whose distribution can be found in Figure A1d, the lost information of fields in Figure A1e can also be successfully recovered, as depicted in Figure A1f.

**Figure. A1** (a) The random distribution of conductivity $\sigma$ between 0.08 S/m and 0.12 S/m. (b) Simulated $\text{E}_{\text{z}}$-distribution at $f=\text{11 GHz}$ for random conductivity distribution as in (a). (c) The restored $\text{E}_{\text{z}}$-distribution at the complex frequency $f=\text{(11+0.899i) GHz}$. (d) The random distribution of conductivity $\sigma$ between 0.07 S/m and 0.13 S/m. (e) Simulated $\text{E}_{\text{z}}$-distribution at frequency $f=\text{11}\text{.5}\text{ GHz}$ for random conductivity distribution as in (d). (f) The restored $\text{E}_{\text{z}}$-distribution at the complex frequency $f=\text{(11.5+0.899i) GHz}$.

**3.** **Loss compensation for SPPs at an interface between air and the Drude model metal.**

Here, we provide a detailed derivation to demonstrate the process of obtaining complex frequency for strongly dispersive media. We assume the relative permittivity of metal is described by the Drude model, i.e., $\epsilon_{m}=\epsilon_{r}-\omega_{p}^{2}/(\tilde{\omega}^{2}-i\tilde{\omega}\gamma)$, where $\epsilon_{r}$, $\omega_{p}$and $\gamma$ represent the constant at infinite frequency, plasma frequency and dissipation term, respectively. Thus, under the excitation of complex frequency, $\tilde{\omega}=\omega_{r}+i\omega_{i}$, the wavevector of SPP is $\tilde{k}=\tilde{\omega}\sqrt{\epsilon_{m}}/(c\sqrt{1+\epsilon_{m}})$, which can be expressed as

$\tilde{k}^{2}=\frac{\left( \omega_{i}-i\omega_{r} \right)^{2}\left( -\omega_{p}^{2}-\epsilon_{r}\left( \omega_{i}-i\omega_{r} \right)\left( \omega_{i}-\gamma-i\omega_{r} \right) \right)}{c^{2}\left( \omega_{p}^{2}-\left( \epsilon_{r}+1 \right)\left( \omega_{i}-i\omega_{r} \right)\left( \omega_{i}-\gamma-i\omega_{r} \right) \right)}$,	(A6)

in order to achieve lossless propagation, the imaginary part of $\tilde{k}^{2}$ should be zero. Considering $\omega_{r}\gg\omega_{i}$ and $\omega_{p}\gg\gamma$, we can simplify Eq.A6 by omitting the high-order terms, the imaginary part of the imaginary part of $\tilde{k}^{2}$ can be further given by

$Im\left( \tilde{k}^{2} \right)\approx\frac{2\omega_{i}\epsilon_{r}\left( 1+\epsilon_{r} \right)\omega_{r}^{5}-\left( \gamma+4\omega_{i}\epsilon_{r} \right)\omega_{r}^{3}\omega_{p}^{2}+2\omega_{i}\omega_{r}\omega_{p}^{4}}{\left( 1+\epsilon_{r} \right)^{2}\omega_{r}^{2}\left( \left( 2\omega_{i}+\gamma\right)^{2}+\omega_{r}^{2} \right)-2\left( 1+\epsilon_{r} \right)\omega_{r}^{2}\omega_{p}^{2}+\omega_{p}^{4}}$,	(A7)

By solving $Im\left( \tilde{k}^{2} \right)=0$, the complex frequency corresponding to lossless propagation of SPP is expressed as

$\omega_{i}\approx\frac{\gamma\omega_{r}^{2}\omega_{p}^{2}}{2\left( \epsilon_{r}\omega_{r}^{4}+\epsilon_{r}^{2}\omega_{r}^{4}-2\epsilon_{r}\omega_{r}^{2}\omega_{p}^{2}+\omega_{p}^{4} \right)}$,	(A8)

By assuming $\epsilon_{r}=5$, $\omega_{p}=2\pi\times2.5\times{10}^{3} \mathrm{THz}$ and $\gamma=0 \mathrm{THz}$, the dispersions of SPP and light line can be found in **Figure A2**, it is shown that the dispersion of SPP is below that of light line, indicating that such metal can support SPPs below $\omega_{p}/\sqrt{5}$. To verify this, we calculate field distribution of SPP by using COMSOL Multiphysics, as shown in **Figure A3**a, when $\gamma=0 \mathrm{THz}$, lossless propagation of SPP can be successfully observed, but for $\gamma=2\pi\times2\times10 \mathrm{THz}$, SPP will decay during propagation along interface under real frequency excitation, which is shown in Figure A3b, and the enlarged view of red line box is shown in Figure A3c. By calculating Eq. A8, we can obtain $\omega_{i}\approx6.23 \mathrm{THz}$ for $\omega_{r}=900 \mathrm{THz}$. Utilizing the magnetic field with a frequency range from 820 THz to 980 THz and a frequency spacing of 0.2 THz, the lost field information of SPP can be restored and SPP at 900+6.23i THz propagates at the interface without dissipations, illustrated in Figure. A3d.

**Figure. A2** The dispersion of SPP at an interface between air and a strongly dispersive metal.

**Figure. A3** (a) SPP excitation at the interface between air and lossless metal. (b) SPP excitation at the interface between air and lossy metal. (c) the enlarged view of the red box in (b). (d) field distribution for $\tilde{\omega}=900+6.23i \mathrm{THz}$ under virtual complex frequency wave excitation.

**4. The theoretical model of the coherent virtual absorption**

As shown in **Figure A4**a, we start with the analytical model to investigate the transmission and reflection properties of the system composed of a transmission channel and a bright resonator. According to the CMT, assuming a time-dependent term $e^{i\tilde{\omega}t}$, we can establish the master equations

$\frac{da}{dt}=\left[ i\omega_{0}-\Gamma-\gamma\right]a+i\sqrt{\kappa}s_{1,in}$,	(A9a)

$s_{2,out}=s_{1,in}+i\sqrt{\kappa}a$,	(A9b)

$s_{1,out}=i\sqrt{\kappa}a$,	(A9c)

where $\omega_{0}$, $\gamma$, $\Gamma,$ and $\sqrt{\kappa}$ denote the resonance frequency, radiation loss, non-radiation loss, and the coupling strength between the transmission channel and resonator, respectively. From Equation A6a-c, the transmission and reflection coefficients can be derived as

$t=1+\frac{-\kappa}{\left( i\tilde{\omega}-i\omega_{0}+\Gamma+\gamma\right)}$,	(A10a)

$r=\frac{-\kappa}{\left( i\tilde{\omega}-i\omega_{0}+\Gamma+\gamma\right)}$,	(A10b)

By applying $\kappa=\gamma$ and $t+r=0$, we can obtain the condition to induce coherent perfect absorption (CPA), which is expressed as

$\tilde{\omega}=\omega_{0}-i\left( \gamma-\Gamma\right)$,	(A11)

where it is found that $\gamma=\Gamma$ indicates conventional CPA with real-frequency incident waves [32,36]. Considering a general case with $\gamma\neq\Gamma$, CPA shall occur at complex frequencies, which is called coherent virtual absorption. Here, the real and imaginary parts of complex frequencies to realize CPA are determined by the resonance frequency of the bright resonator and the difference between radiation loss $\gamma$ and non-radiation loss $\Gamma$, respectively. To explore the transmission and reflection properties, as shown in Figure A5b, we numerically calculate $\left| \text{t+r} \right|$ as a function of real frequency under different non-radiation losses, i.e., $\text{Γ}\text{ }\text{=}\text{ }\text{0.1, 0.25, 0.5, 0.75}$, with a fixed radiation loss $\text{γ}\text{ }\text{=}\text{ }\text{0.5}$. It is found that the conventional CPA occurs only at $\Gamma=\gamma$, agreeing well with our previous analysis.

**Figure. A4** (a) Schematic diagram of the theoretical model composed of a bright resonator and transmission channel, where $\omega_{0}$, $\gamma$, $\Gamma,$ and $\kappa$ denote the resonance frequency, radiation loss, non-radiation loss, and the coupling strength between the transmission channel and resonator, respectively. (b) The calculated $\left| t+r \right|$ as a function of real frequencies with different non-radiation loss $\Gamma=0.1, 0.25, 0.5, 0.75$, where a fixed radiation loss $\text{γ}\text{ }\text{=}\text{ }\text{0.5}$ is considered. The resonance frequency is $\text{ω}_{\text{0}}\text{=1.6}$. (c) The calculated transmission and reflection coefficient for $\Gamma=0.1$ with respect to $Re(\tilde{\omega})$. (d) The calculated $\left| t+r \right|$ for $\Gamma=0.1, 0.2, 0.3, 0.4, 0.5,-0.1$ as a function of complex frequency $\tilde{\omega}$.

Next, we demonstrate that using virtual CFW excitation can obtain the transmission and reflection coefficients at complex frequencies. Taking $\Gamma=0.1$ of Figure A5b for example, we plot the transmission (green line) and reflection (navy line) for real frequency in **Figure A4**c, which can be depicted by the 15-order polynomial, which are given by

$t\left( \omega\right)=a_{0}+a_{1}\omega+a_{2}\omega^{2}+\ldots+a_{15}\omega^{15}$,	(A12a)

$r\left( \omega\right)=b_{0}+b_{1}\omega+b_{2}\omega^{2}+\ldots+b_{15}\omega^{15}$,	(A12a)

where ${a_{0},\ldots,a}_{15}$ and $b_{0},\ldots,b_{15}$ are coefficients of polynomials. Here, by bringing the complex frequency $\tilde{\omega}$ with an imaginary part $\omega_{i}=-0.4i$ into Equation A12a-b, the transmission (blue circle) and reflection (green circle) for complex frequencies are shown in Figure A4c, which are identical to those obtained by analysis of Equation A10a and b corresponding to red and black lines. Moreover, the amplitude of the transmission and reflection coefficient is equal at $Re\left( \tilde{\omega} \right)=1.6$, indicating that virtual CPA shall occur at $\tilde{\omega}=1.6-0.4i$, as shown in Figure A4d, where other different non-radiation losses, i.e., $\Gamma=\text{ 0.2, 0.3, 0.4,}\text{ }\text{-0.1}$, are considered, coherent perfect absorption can also be achieved.

Finally, in order to demonstrate the effects of amplitude and phase imbalance between two inputs, we define amplitude ratio *A* and phase difference $\varphi$ of two inputs, and analytically calculate $|t+r|$ as a function of amplitude ratio and phase difference, as displayed in Figure. A5a, the minimum value of $|t+r|$ occurs at *A*=1 and $\varphi=0$, indicating coherent virtual absorption requires equal amplitude and in-phase waves, which is also confirmed by experimental results in Figure. A5b. So, even with the incorporation of complex frequencies, the coherence necessity to induce coherent perfect absorption remains.

**Figure. A5** (a) analytically calculated $|t+r|$ as a function of amplitude ratio and phase difference. (b) experimentally measured $|t+r|$ as a function of amplitude ratio and phase difference.

**5. Comparison between polynomial fitting and rational fitting**

In our work, we only use polynomial fitting to construct the bridge linking complex and real frequencies. However, we also find that using polynomial fitting will fail to process resonances with very narrow linewidths. Other fitting functions, i.e., Lorentzian fitting or rational fitting, shall be considered instead. For example, in the section on experimental coherent virtual absorption, transmission coefficients derived from coupled-mode theory can be written as

$t=1+\frac{-\gamma}{\left( i\tilde{\omega}-i\omega_{0}+\Gamma+\gamma\right)}$,	(A12)

which is provided in section 4 of the Supporting Information. where, $\Gamma=0$ and $\omega_{0}=1.5$ are used. Obviously, it is a resonant behavior and radiation loss $\gamma$ determines resonant linewidth, as shown in **Figure A6**a and c, under real frequency excitation, the analytically calculated transmission coefficients for $\gamma=0.5$ and $\gamma=0.05$ clearly show that a smaller radiation loss results in a narrower resonant linewidth. Further, we use both polynomial fitting and rational fitting to describe transmission coefficients, where rational fitting is expressed as $F\left( \omega\right)=\sum_{k=1}^{20} \frac{C_{k}}{\omega-\omega_{k}}+D$, and $c_{k}$, $\omega_{k}$ and $D$ denote residues, poles and direct term, respectively. As shown in Figure A6a, for $\gamma=0.5$, both polynomial fitting and rational fitting align well with the analytical calculation, and the corresponding transmission coefficients at complex frequencies can be found in Figure A6b by inserting the complex frequencies with an imaginary part of $\omega_{i}=0.1$ into the expressions obtained by polynomial and rational fitting, exhibiting a good agreement with analyses. But for $\gamma=0.05$ in Figure. A6c, the polynomial fails even if the order of the polynomial is increased to 100, eventually leading to an incorrect transmission coefficient at complex frequencies in Figure. A6d. On the contrary, rational fitting is still effective to obtain the transmission coefficient at complex frequencies, as displayed in Figure. A6d, indicating that rational fitting has advantages in processing resonance with a very narrow linewidth.

However, rational fitting will fail in some simple cases. For example, when fitting the electric field of a plane wave, i.e., $E_{z}=sin(\psi)$, here, $\psi$ refers to the propagating phase, given by $\psi=2\pi fd$, here $f$ and $d$ denote frequency and position. In **Figure A7**, it is obviously shown that using rational fitting leads to wrong results.

In summary, we should choose the appropriate fitting methods, such as polynomial fitting, rational fitting, etc., according to the properties of electromagnetic responses.

**Figure. A6** (a) analytically calculated transmission coefficient, polynomial fitting and rational fitting at real frequencies under $\gamma=0.5$, where black line, blue ring and red pentagram represent analytical calculation, polynomial fitting and rational fitting, respectively. (b) analytically calculated transmission coefficient, polynomial fitting and rational fitting at complex frequencies with fixed imaginary part of $\omega_{i}=0.1$ under $\gamma=0.5$. (c) analytically calculated transmission coefficient, polynomial fitting and rational fitting at real frequencies under $\gamma=0.05$. (d) analytically calculated transmission coefficient, polynomial fitting and rational fitting at complex frequencies with a fixed imaginary part of $\omega_{i}=0.1$ under $\gamma=0.05$.

**Figure. A7** Polynomial fitting and rotational fitting for the electric field of a plane wave.
